# Setup errors in radiation therapy for thoracic tumor patients of different body mass index

**DOI:** 10.1002/acm2.12270

**Published:** 2018-03-01

**Authors:** Jin Zhao, Mingyun Zhang, Fushan Zhai, Haiyan Wang, Xingde Li

**Affiliations:** ^1^ Department of Oncology Cangzhou Central Hospital Cangzhou Hebei China; ^2^ Department of Radiation Oncology Third Hospital of Hebei Medical University Cangzhou Hebei China; ^3^ Department of Radiation Oncology Cangzhou Central Hospital Cangzhou, Hebei China

**Keywords:** body mass index (BMI), radiation therapy, setup errors, thoracic tumor

## Abstract

**Purpose:**

To assess the setup errors in radiation therapy for thoracic tumors patients of different somatotypes, and to seek an individualized mathematical basis for defining the planning target volume (PTV).

**Methods:**

Sixty patients with thoracic tumors were divided into four somatotypes according to their body mass index (BMI), and their body positions were setup by two groups of technicians independently. CT simulations were performed and the reconstructed radiography was digitally generated as reference images for location verification and error measurement. By setting positioning error ranges, the within‐range positioning correction rate was compared among groups.

**Results:**

Position setups for patients in the emaciated group, moderate group, and overweight group were relatively stable (with minor setup error differences between the two groups of technicians). In emaciated group, moderate group, overweight group, and obese group, setup errors in the right–left direction (R‐L) were 2.2 ± 1.3 mm, 2.2 ± 1.6 mm, 3.9 ± 3.1 mm, and 8.8 ± 3.5 mm, respectively; whereas the setup errors in the four groups in the superior–inferior(S‐I) direction were 2.4 ± 1.8 mm, 2.1 ± 1.9 mm, 3.2 ± 2.6 mm, and 5.4 ± 3.5 mm, and in the anterior–posterior (A‐P) direction were 2.2 ± 1.7 mm, 1.9 ± 1.9 mm, 3.2 ± 2.9 mm, and 6.2 ± 4.2 mm, respectively. Moreover, in the moderate group, the positioning correction rate in the three directions (R‐L, S‐I, and A‐P) was 20%, 9%, 8% within the error range of 5–10 mm, and 3%, 0%, 1% with a more than 10 mm error range. However, in overweight group and obese group, the positioning correction rate in these three directions (also with a more than 10 mm error range) was 23%, 27%, 19% and 21%, 16%, 23%, respectively.

**Conclusions:**

In radiation therapy for patients with thoracic tumors, the definition of PTV should be individualized. Meanwhile, with the increase in BMI, positioning correction rate has a tendency to rise too.

## 1 INTRODUCTION

With the development of radiotherapy, intensity‐modulated radiation therapy (IMRT) has become the mainstream method in modern radiotherapy technology with its obvious dosimetric advantage.^1^, ^2^, ^3^ The accurate positioning and setup are needed with the increasing use of IMRT. Due to the factors such as respiratory, body weight, skin traction, arm lift, etc, the repeatability of whole‐treatment process is poor.^4^ There are a variety of factors influencing the setup errors in radiation therapy for patients with thoracic tumors.^5^, ^6^, ^7^ In order to reduce these setup errors, many researchers tried to improve setup accuracy by improving the fixation of patients.^8^, ^9^, ^10^ In the study of Wang Wei etc.,^11^ it was shown that BMI was positively correlated with total error, indicating that BMI was an important factor in the setup errors. Yet there was scarcely any studies regarding whether there were significant differences in the setup error magnitude under the same fixation conditions among patients of different somatotypes. Our study measured the setup errors in radiation therapy for thoracic tumors patients of different somatotypes, and compared the impact of positioning correction rate on setup accuracy within different error ranges. And we aim to provide evidence for the patient‐specific definition of PTVs in radiation therapy.

## 2 MATERIAL AND METHODS

### 2.A General clinical data

Sixty patients (30 male and 30 female, age ranges from 38 to 78 yr old, with an average age of 52.6 yr old) who underwent IMRT in our hospital from December 2011 to March 2013 were enrolled in this study. Of all these patients, 30 cases (50.0%) were with lung cancer, 23 cases (38.3%) with esophageal carcinoma and seven (11.7) with mediastinal tumor.

### 2.B Height, weight, body mass index (BMI), and grouping

Height and body weight of the patients were measured according to the method recommended in the Guidelines for Prevention and Control of Overweight and Obesity in Chinese Adults in 2004, and their BMIs were calculated.^12^ Then patients were divided into four groups according to Chinese standard based on their BMI value: patients with BMI < 18.5 kg/m^2^ were allocated into the emaciated group, patients with BMI within the range of 18.5–24.0 kg/m^2^ were allocated into moderate group, with BMI between 24.0–28.0 kg/m^2^ were assigned into overweight group, whereas patients with BMI ≥ 28 kg/m^2^ were grouped into the obesity group. And there were 15 patients in each group finally.

### 2.C Equipment and materials

Varian Clinac CX linear accelerator was used for the radiotherapy treatment. Eclipse planning system (Varian Inc., Palo Alto, CA, USA) was used for the formulation of various radiation treatment plans. The 64‐slice CT simulator (Siemens, Munich, Germany) and all‐digital X‐ray simulator (Shandong Xinhua, China) were used for simulation positioning and planning validation. And we used the fixed body frame (MEDTEC, USA), thermoplastic sheet (MEDTEC, USA) and electrode paste (type I, Hangzhou Tianyi Medical Devices Co., Ltd.) to fix patients' position.

### 2.D Positioning and measurement method of setup errors and definition of positioning correction rate

Patient took a supine position on the body frame, and was fixed with a spoon‐shaped headrest, with their hands placed on the forehead cross‐armed. A thermoplastic sheet was taken out from the thermostatic water tank and spread evenly on the patient body surface while fixed onto the body frame. After cooling, the shaped sheet was removed to drill three non‐collinear holes (with diameters of about 5 mm, which was the same size of the metal head of the electrodes) where the sheet was closest to the skin (indicating small skin movement). Then it was again covered on the patient and the locations of the three holes were marked to attach electrode paste (no skin allergies were found in our study). Before treatment, the holes on the sheet would be aligned with the metal head of the electrode paste by technicians, and patient position was adjusted with the sagittal laser line so the sheet was fixed naturally. Before treatment, there were five times of consecutive validation for each patient, and setup was completed under simulator by two independent groups of technicians. Then a total of 300 sets of data were obtained from the AP and lateral validation images along the central axis. Because of the good visibility of bony structures on the 2D images, the repeatability and stability were better, so the sternum or vertebral body closest to the tumor was selected as reference point. The distance from the reference point to each boundary of the radiation field was measured. Then the absolute value of the difference of measurement‐on‐planning‐system minus measurement‐on‐simulator‐validation‐images was taken as the setup errors during repeating positioning. Thus the mean value of setup errors in the three directions (R‐L, A‐P, and S‐I) was calculated. The determination of reference points and the measurement of actual distance were completed by an attending doctor. The error range was set between 3 and 10 mm, which was divided into four groups (≤ 3 mm, 3–5 mm, 5–10 mm, and > 10 mm), and the positioning correction rate within different error range was determined and compared. Re‐positioning would be required if setup errors exceeded error range. Positioning error correction rate within certain error range was defined as the percentage of patients that needed re‐positioning in each group.

### 2.E Statistical analysis

SPSS 17.0 software was used for statistical analysis. And group *t*‐test or one‐way ANOVA was used in comparison between groups, whereas LSD test was used in pairwise comparisons between groups. *P* < 0.05 indicates statistical significance.

## 3 RESULTS

### 3.A Comparisons between the positioning by two groups of technicians

Only the three‐dimensional positioning errors in the obesity group showed statistically significant difference between the two groups of technicians (Table 1).

**Table 1:** Set‐up errors in the four somatotypes by different technicians (mm)

Group	Emaciated group	Moderate group	Overweight group	Obesity group
Technicians of group A	4.7 ± 4.4	5.0 ± 4.4	5.9 ± 5.4	10.1 ± 4.0
Technicians of group B	3.5 ± 1.9	4.8 ± 3.5	5.0 ± 4.2	15.1 ± 7.6
*T* value	0.31	0.55	0.21	2.26
*P* value	0.78	0.69	0.81	0.043

### 3.B Impact of BMI on setup errors

When compared with the moderate group, the emaciated group showed no significant difference in patient setup errors. There was significant difference between overweight group and obesity group for setup errors, and the value was significant difference in R‐L direction in the obesity group. The similar conclusions were drawn in the other two directions. See Table 2.

**Table 2:** Comparisons of set‐up errors among the four somatotypes (mm)

Group	R‐L direction	C‐C direction	A‐P direction
Emaciated group	2.2 ± 1.3	2.4 ± 1.8	2.2 ± 1.7
Moderate group	2.2 ± 1.6	2.1 ± 1.9	1.9 ± 1.9
Overweight group	3.9 ± 3.1	3.2 ± 2.6	3.2 ± 2.9
Obesity group	8.8 ± 3.5	5.4 ± 3.5	6.2 ± 4.2
*F* value	15.77	3.41	4.56
*P* value	0.000	0.046	0.028

### 3.C Positioning correction rate in the four somatotypes

The positioning correction rate of moderate group in the three directions (R‐L, A‐P, and S‐I) within different error range was shown (20%, 9%, and 8% within the error range of 5–10 mm, and 3%, 0%, 1% within the error range of > 10 mm) in Table 3. The results in overweight group and obesity group showed two sets of positioning correction rate of 23%, 27%, 19% and 21%, 16%, 23%, respectively (within an error range of > 10 mm, in the order of R‐L, A‐P, and S‐I). This suggested that for 97% of patients in the emaciated group and moderate group, an estimated CTV‐PTV margin of 10 mm was quite enough to make up for the setup errors generated by daily positioning. However, in the overweight group and obesity group, even with an estimated CTV‐PTV margin of 10 mm, there were still 27% of the patients needing positioning correction in the R‐L direction, and 21% needing correction in the A‐P direction, and 23% in the S‐I direction.

**Table 3:** Positioning correction frequency of the four somatotypes (%)

Error range (mm)	Emaciated group			Moderate group			Overweight group			Obesity group		
	R‐L	A‐P	C‐C	R‐L	A‐P	C‐C	R‐L	A‐P	C‐C	R‐L	A‐P	C‐C
≤3	62	74	76	59	78	79	98	96	90	100	100	100
3˜5	17	15	14	18	13	12	20	15	14	23	12	24
5˜10	18	9	10	20	9	8	55	62	60	50	67	53
>10	3	2	0	3	0	1	23	19	16	27	21	23

### 3.D Correlation between positioning correction rate and error range in the four somatotypes

**Figure 1:**
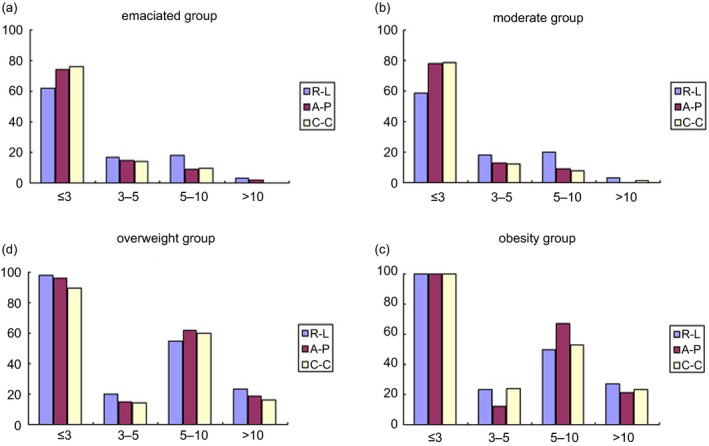
(a) Relationship between the correction rate and error range for emaciated group. (b) Relationship between the correction rate and error range for moderate group; (c) Relationship between the correction rate and error range for overweight group; (d) Relationship between the correction rate and error range for obese group.

Figure 1 showed that positioning correction rate in the emaciated group and moderate group was mainly concentrated on the error range of 5 mm, whereas no such central tendency was seen in overweight group or obesity group. This result indicated that for patients with BMI < 24 kg/m^2^, an estimated CTV‐PTV margin of at least 5 mm was enough to avoid obvious setup errors in most cases (specifically more than 77% in the R‐L direction, 89% in the A‐P direction, and 90% in the S‐I direction). On the other hand, for patients with BMI ≥ 24 kg/m^2^, the estimated CTV‐PTV margins should be individualized.

## 4 DISCUSSION

The purpose of setup before treatment is to repeat the patient position set by simulator, so as to repeat the PTV and the spatial relationship between radiation field and organs‐at‐risk, which was determined during previous planning.^13^ Therefore it can ensure accurate beam irradiation on the target.^14^ Hunt et al. thought that precise treatment was more affected by position error compared with conventional radiotherapy.^15^ The dose distribution which is formulated by plan design is only an ideal model. There is a large gap between actual dose distribution and planned dose distribution because of the existence of position error. Therefore, a higher requirement is needed for the position of precise radiotherapy. By analyzing the influence of patient's own body mass index on the position error, we expect to avoid the plan which is based on the general population data, so as to obtain the individualized treatment plan.

In the study of Kutcher GJ etc.,^16^ it was shown that the thickness of subcutaneous fat layer, muscle tension, gravity, postural comfort, self‐control ability of patients and other factors would affect the reproducibility of patient setup. BMI, calculated as weight (kg)/height^2^ (m^2^),^17^ is an objective indicator which can roughly estimate patients' somatotype and indirectly reflect the thickness of subcutaneous fat layer. In this study, we divided the patients into four groups according to their own body mass index. First, we analyzed the influence of BMI on the position error in different technician groups. We found that for patients in the emaciated group, moderate group, and overweight group, patients' setup by two independent technician groups was stable, with no significant difference was found in setup errors. Significant difference of setup errors by different technicians appeared only in the obesity group. The above results indicated that the positioning of our technicians was quite stable, and that setup errors were mainly associated with patients' somatotypes.

It was also shown in our study that setup errors in the three directions (R‐L, A‐P, and S‐I) were similar between the emaciated group and moderate group, whereas setup errors in overweight group and obesity group were significantly greater than that in moderate group, especially in the R‐L direction. In addition, there was an increase trend of setup errors, or decrease trend of setup reproducibility with the increase in BMI. The possible reasons were as follows: (a). Due to the shifting of thick subcutaneous fat in overweight and obese patients in supine position, the markers on patient's skin surface was also displaced; (b). Self‐control ability of overweight and obese patients was relatively weaker than that of patients with lean or moderate body figure; (c). The electrode paste used in our study formed three projections on the barrel‐shaped chest of the patient, which increased setup accuracy but produced the following disadvantage during patient positioning: the force of the technicians may not balance on both sides of the patient (most likely happens in patients with thick subcutaneous fat layer), so that setup errors occurred easily, particularly in the R‐L direction.

Furthermore, our results demonstrated that in the emaciated group and moderate group, most setup errors (more than 77% in the R‐L direction, 89% in the A‐P direction, and 90% in the S‐I direction) were within the range of 5 mm, and the positioning correction rate of error range > 10 mm dropped to less than 3%. On the other hand, in the overweight group and obesity group, with error range > 10 mm, the positioning correction rate in the three directions reached 23%, 27% (R‐L), and 19%, 19% (A‐P), and 16%, 23% (S‐I), respectively. These results suggested that during the definition of PTV for patients with BMI < 24 kg/m^2^, the estimated margins should be at least 5 mm. However, it should be noted that when there were endangered organs around, the estimated margin in the R‐L direction should be expanded to 10 mm, for most setup errors greater than 5 mm would occur in this direction. And while there was significant decrease in the positioning correction rate of errors greater than 10 mm, a 10 mm margin would be enough to make up for about 97% of the setup errors generated by daily operation. Yet for patients with BMI ≥ 24 kg/m^2^, especially for those with BMI ≥ 28 kg/m^2^, there were still 27% of the patients who need positioning correction in the R‐L direction, 21% in the A‐P direction, and 23% in the S‐I direction, even with an estimated CTV‐PTV margin of 10 mm. In such cases, an individualized online correction would be necessary. Previous studies proposed adaptive radiotherapy for each patient in the early stage of the whole course of radiotherapy.^18^, ^19^ They measured each of the setup errors, conducted statistical analysis, so as to determine whether to modify the radiotherapy plan or not. It is very expensive to carry out image guided radiation therapy, and the treatment cost is high. At present, there is still a big gap between China's radiotherapy machine and the other countries',^20^ and not all medical institutions have such equipment. Therefore, we can screen out the patients who are in need of the individualized radiotherapy plan by BMI from the perspective of evidence‐based medicine, in order to improve the accelerator utilization, reduce unnecessary human and material resources and related treatment cost. However, because of the small sample size of this study, we need to expand the sample size in clinical work for further observation and analysis so as to find the best cost‐benefit population by BMI.

However, there were some limitations in our study. First, we performed the five consecutive setup validations for each patient. We must acknowledge that the limitation of this design was that the five consecutive validation pairs were unlikely to capture inter‐fractional and large intra‐fractional setup variations. Second, the results were not applicable if daily imaging‐based setup is used for all patients even with conformal curative therapy due to the resource limitation. Therefore, our results were useful for allocating the imaging resources to the patients who would benefit the most.

In summary, for tumor patients with different BMIs, it was not enough to adopt a uniform PTV margin. And it should be adjusted based on the individual conditions of each patient. Moreover, BMI could be used to screen out patients who need individually adapted radiotherapy, so that the utilization of the accelerator could be improved, and unnecessary cost of manpower, material, and other related treatment expenses could be reduced.

## CONFLICT OF INTEREST

The authors declare that there is no conflict of interests to be disclosed.

All patients were informed consent and this study was carried out by the approval of the Hospital Ethics Committee.

## Supporting information


**Figure S1.** The locations of holes drilled into the patient thermoplastic mask.

**Figure S2.** The locations of holes drilled into the patient thermoplastic mask.
