# Supplementary figures and images for: Setup errors in radiation therapy for thoracic tumor patients of different body mass index
Source: J Appl Clin Med Phys. 2018 Mar 1;19(3):27–31. doi: 10.1002/acm2.12270 (PMC5978940; doi:10.1002/acm2.12270)

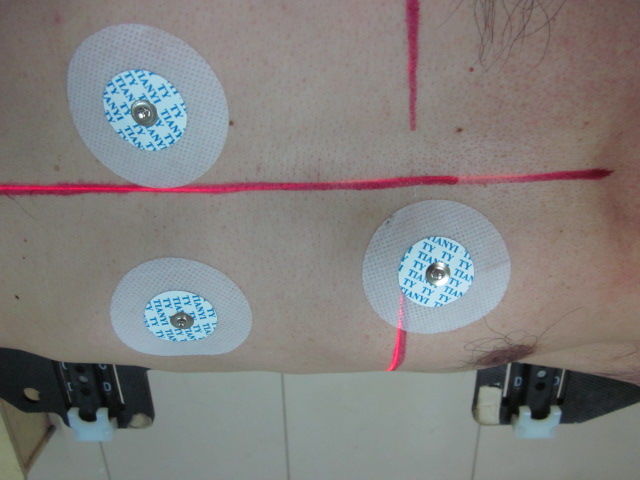

Supplement: Supplementary file 1 — Figure S1. The locations of holes drilled into the patient thermoplastic mask. [file ACM2-19-27-s001.jpg]

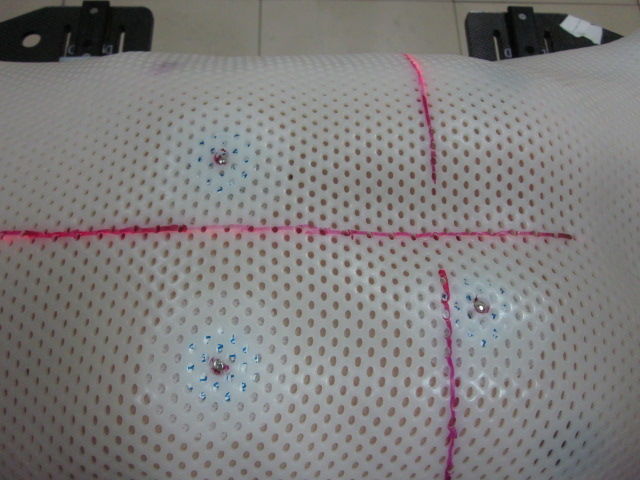

Supplement: Supplementary file 2 — Figure S2. The locations of holes drilled into the patient thermoplastic mask. [file ACM2-19-27-s002.jpg]
